# Basal Cell Adenoma of the Upper Lip: Report of a Rare Case With Literature Review

**DOI:** 10.7759/cureus.52599

**Published:** 2024-01-19

**Authors:** Syed A Ahmad, Deepika B Popli, Keya Sircar, Shamimul Hasan

**Affiliations:** 1 Oral and Maxillofacial Surgery, Faculty of Dentistry, Jamia Millia Islamia, New Delhi, IND; 2 Oral and Maxillofacial Pathology, Faculty of Dentistry, Jamia Millia Islamia, New Delhi, IND; 3 Oral Medicine and Radiology, Faculty of Dentistry, Jamia Millia Islamia, New Delhi, IND

**Keywords:** histopathology, benign oral mass, minor salivary gland, upper lip, basal cell adenoma, salivary gland tumor

## Abstract

Basal cell adenoma (BCA) is a rare, benign tumor originating from the epithelial cells of the salivary glands. It was earlier categorized as a subtype of monomorphic adenoma with distinctive histopathological features. BCA usually manifests as asymptomatic, slow-growing masses that exhibit a site and age predilection, commonly affecting the major salivary glands of elderly female patients. Histologically, solid, trabecular, tubular, and membranous patterns are recognized. It is imperative to establish a precise distinction between BCA, pleomorphic adenoma, and malignant salivary gland tumors before initiating treatment to ensure effective management. The standard treatment approach is surgical resection of the tumor. Recurrence and malignant transformation rarely occur, except for the membranous subtype.

This article aims to report an unusual case of BCA arising from a minor salivary gland in the upper lip. The post-operative course was unremarkable, with complete healing of the surgical site. No recurrence was observed during a one-year follow-up. BCA arising from a minor salivary gland in the upper lip is an extremely uncommon entity. A comprehensive review of BCA in the upper lip, reported from 1991 to December 2023, revealed only 14 cases.

## Introduction

Basal cell adenoma (BCA) is a benign salivary gland tumor, typically originating in the parotid gland. It is distinguished by the basaloid morphology of the tumor cells and the lack of the myxochondroid stromal component [1]. Basal cell adenomas are infrequently encountered and comprise 1.5% of all epithelial and 2.4% of all benign salivary gland tumors [2]. The tumor is frequently observed in older individuals, typically occurring in the age range of 50-70 years [3,4]. BCA predominantly affects females (2:1), except for the membranous pattern, which exhibits an even distribution between males and females [3,5]. Histologically, four distinct patterns have been identified: solid, trabecular, tubular, and membranous [1,5-7].

Major salivary glands are primarily affected (75% parotid and 5% submandibular glands), and their occurrence in minor salivary glands is notably rare. When present in minor salivary glands, the primary affected site is the upper lip, followed by the palate, buccal mucosa, and lower lip [1,5-7]. 

Establishing a precise distinction between BCA and other benign and malignant salivary neoplasms like pleomorphic adenoma, adenoid cystic carcinoma (ACC), and basal cell adenocarcinoma (BCAC), is essential for ensuring effective treatment and enhancing the overall prognosis [4,8]. 

The recommended treatment for BCA is conservative excision, and they exhibit a low recurrence rate. BCA rarely exhibits malignant transformation, except for the membranous type, with a 4% rate of malignant transformation [2]. Lasers are considered a standard tool of treatment and care for both patients and surgeons and can be utilized as highly efficient scalpels for the excision of craniofacial tumors with maximum rescue to the vicinity of craniofacial tissues [9,10]. Compared to other salivary gland tumors, BCA exhibits a favorable prognosis, largely due to its infrequent recurrence [8].

## Case presentation

A 48-year-old female patient reported to our OPD for the evaluation of soft tissue mass on the inner aspect of the upper lip. The patient noticed a pea-sized mass four years back, that has gradually progressed to reach its present size. There was no discernible history of trauma or any evidence of factitial lip biting. Medical and family history was non-contributory.

**Figure 1:**
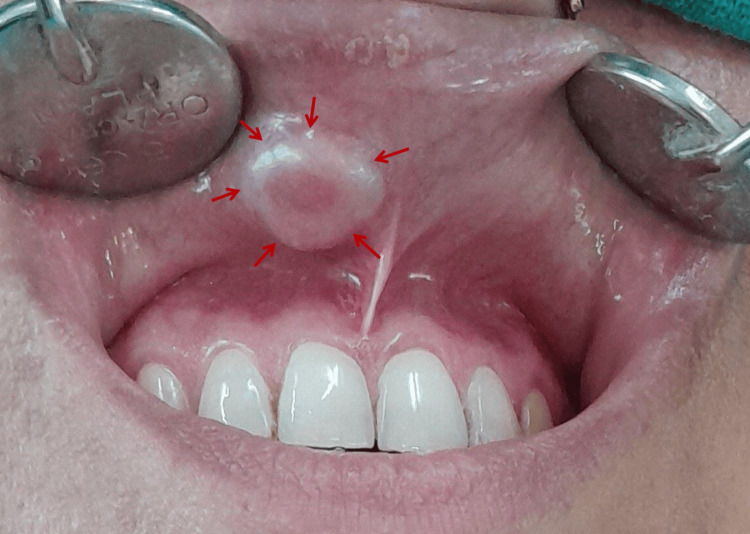
Clinical picture of the upper lip The image is showing a solitary, ovoid soft tissue mass on the right side of the upper lip.

Physical examination was non-contributory, with no nodal enlargement. On intraoral examination, a solitary, ovoid-shaped mass, roughly measuring 1.2 x 1 cm in diameter was observed on the right side of the inner aspect of the upper lip. The mucosa covering the mass was smooth-surfaced, non-ulcerated, and of the same color as the adjacent mucosa. Palpatory findings revealed that the mass was sessile, firm, mobile, non-tender, non-fluctuant, and non-pulsatile. The lesion did not blanch on pressure, and there was no sensory deficit in the lip region (Figure 1).

Considering the anamnesis and clinical appearance of a localized, slow-progressing, smooth-surfaced, well-defined, asymptomatic, freely movable mass located within the upper lip, the lesion was considered of benign origin. Entities of minor salivary gland origin (mucocele, salivary duct cysts, benign and malignant minor salivary gland tumors), infectious origin (odontogenic/non-odontogenic), mesenchymal tumors of adipose tissue (lipoma), vascular entities (hemangioma and lymphangioma), smooth muscle lesions (leiomyoma), or neural derivatives (traumatic neuroma, schwannoma, and neurofibroma) were given a place in the list of differential diagnosis.

**Figure 2:**
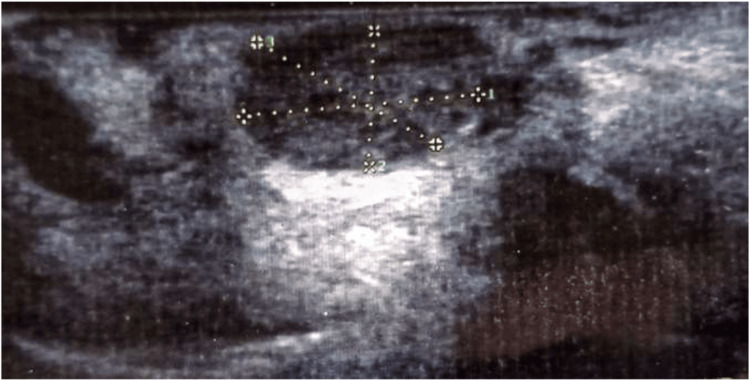
Ultrasonography image The image is showing a well-defined hypoechoic lesion in the upper lip.

Ultrasonography (USG) revealed a well-defined superficial hypoechoic round-ovoid lesion, measuring 8 x 7 x 5 mm with no significant internal and peripheral vascularity site at the upper lip region, possibly suggestive of a benign lesion (Figure 2).

Magnetic resonance imaging (MRI) of the upper lip was advised, but the patient denied the investigation due to financial constraints. MRI would be an excellent aid to rule out malignancy. However, the refusal of MRI did not have any significant impact on the diagnosis and treatment plan, as the history and clinical features were highly suggestive of a benign entity. 

**Figure 3:**
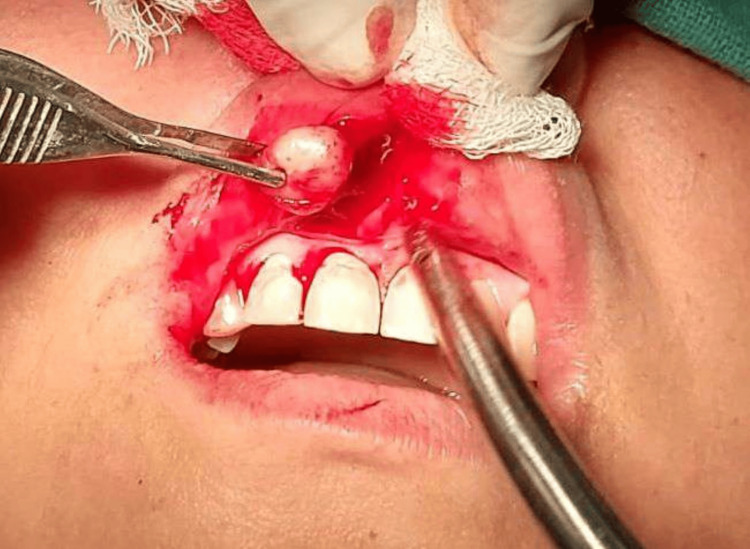
Intra-operative picture showing the complete tumor resection

Hematological investigations, including complete blood count, bleeding, and clotting time, were in the normal range. The treatment procedure was explained to the patient. After obtaining written informed consent from the patient, the complete mass was surgically excised via a blunt horizontal incision of the mucosa with no. 15 blades, taking utmost care not to injure the vital structures, with wide margins under local anesthesia (Figure 3).

**Figure 4:**
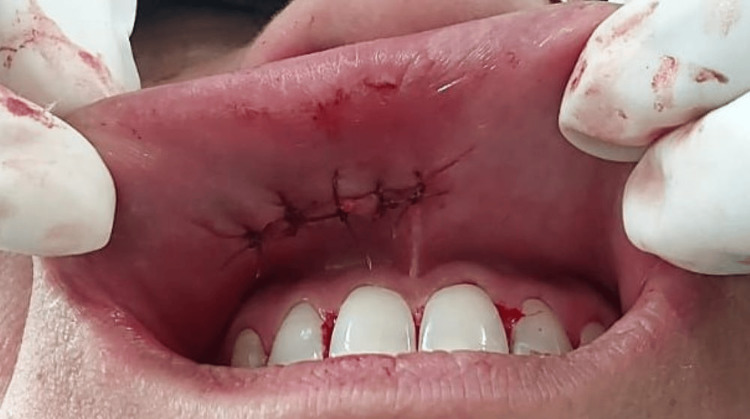
Post-operative picture with the sutures placed

3-0 vicryl was used to suture the muscle bed, and the lip was approximated and sutured (Figure 4).

**Figure 5:**
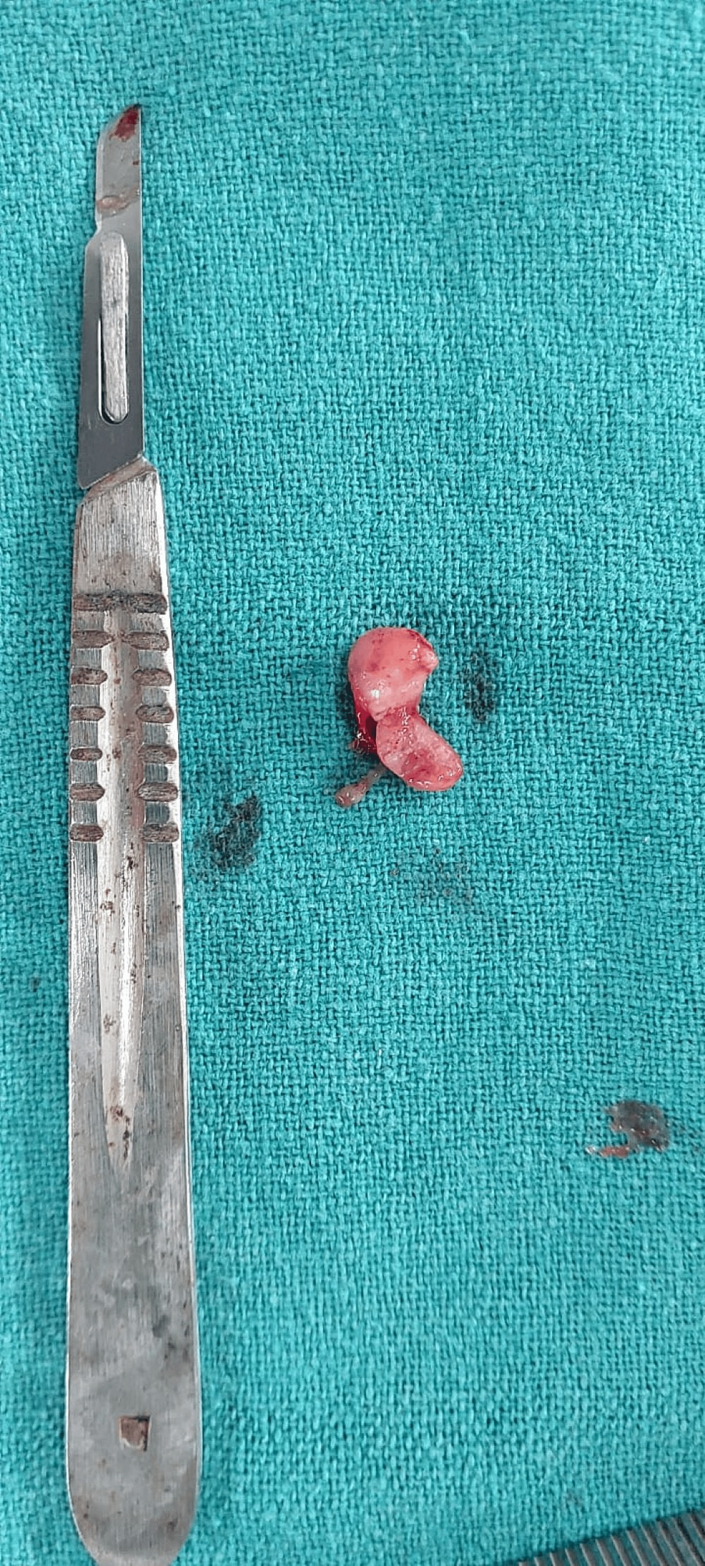
Gross excised lesion specimen

The excised mass, oval in shape, measuring about 1.5 x 1 x 0.5 cm, cream-colored with a smooth surface, and firm in consistency, was sent for histopathologic examination (Figure 5).

**Figure 6:**
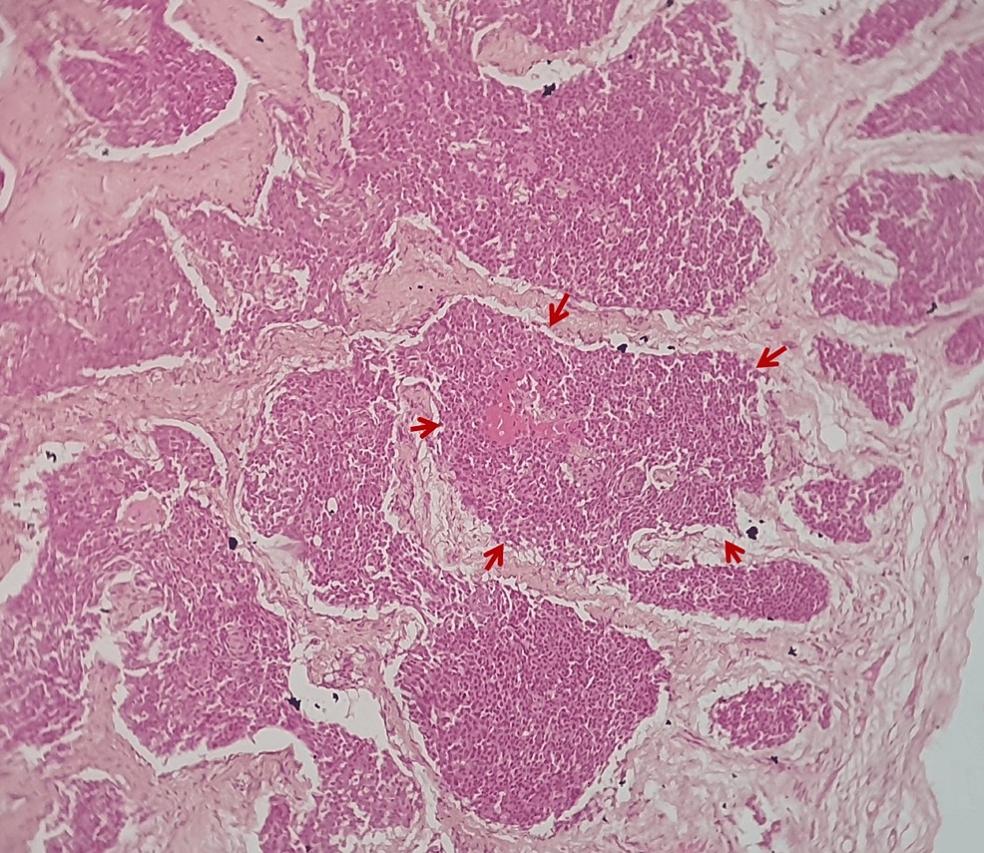
Histopathology image (hematoxylin and eosin stain, 100X) Photomicrograph showing encapsulated tumor with islands of monomorphic basal cells. Red arrows indicate the monomorphic basaloid epithelial pattern.

The histopathological examination showed a well-encapsulated tumor mass composed of monomorphic basaloid cells arranged in the large islands. The submitted hematoxylin and eosin (H & E) stained sections show an encapsulated tumor with large islands composed of basaloid cells with hyperchromatic nuclei (Figure 6).

**Figure 7:**
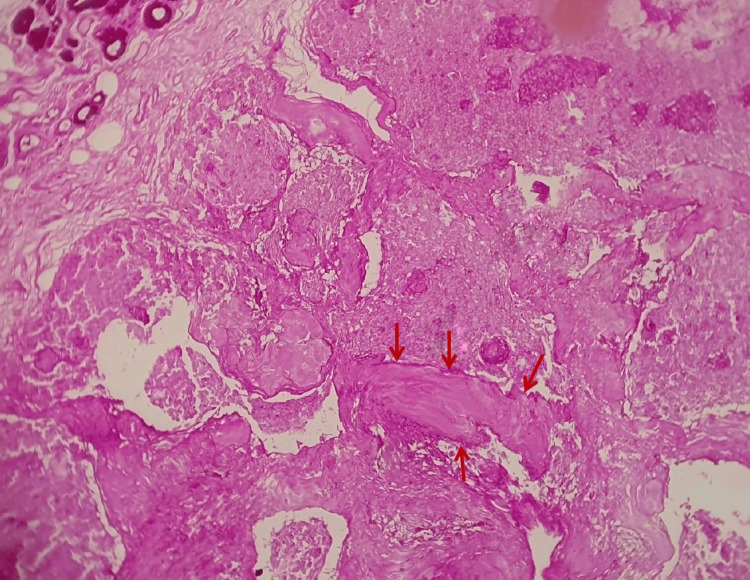
Histopathology image (PAS stain, 100X). Photomicrograph shows that the tumor islands are sharply demarcated from the connective tissue by a prominent basement membrane. Red arrows indicate the sharply demarcated tumor islands by a prominent basement membrane.

The periodic acid-Schiff (PAS) stained section showed a magenta-pink stained basement membrane sharply demarcating the isomorphic tumor islands from the connective tissue. Small cystic spaces with eosinophilic material (PAS positive) were also noted. Mucous salivary acini were also present at the periphery of the lesion (Figure 7).

The histopathological features were consistent with a diagnosis of basal cell adenoma-solid variant. The post-operative course was unremarkable, with complete healing of the surgical site. No recurrence was observed during a one-year follow-up.

## Discussion

In the 1972 World Health Organization (WHO) histological classification of tumors, salivary gland adenomas were divided into two categories: monomorphic and pleomorphic adenomas. According to this classification, basal cell adenoma (BCA) was categorized as a subtype of monomorphic adenoma [11]. Nevertheless, in the 1991 WHO’s classification of salivary gland tumors, the term "monomorphic" has been avoided due to distinct clinicopathological findings that differentiate these entities [12]. Moreover, within this classification, salivary adenomas were sub-classified into nine distinct forms, and BCA was also included in this histological categorization [13].

BCA is a rare benign neoplasm of the epithelial salivary glands, constituting 1-2% of all salivary gland tumors [14]. BCA is generally seen among elderly female patients (mean age of 58 years) and exhibits a site predilection for the major salivary glands, chiefly the parotid gland (80% in the superficial lobe), and the submandibular gland (5%) [15]. Minor salivary gland involvement is an extremely rare occurrence, and the atypical sites for the origin of BCA from minor salivary glands include the upper lip, palate, buccal mucosa, and lower lip [4,8]. Clinically, it manifests as an asymptomatic, slowly progressing, round-ovoid, normal-colored, well-circumscribed, and mobile mass, typically measuring less than 3 cm in diameter [1,3-8]. In 1972, Batsakis was the first to document a case of BCA and proposed that the intercalated duct or basal cell serves as the histogenetic cells [13]. 

BCA was classified as a distinct salivary gland tumor in 1991. A comprehensive manual and electronic literature search was performed using PubMed and Google Scholar search engines employing the following Medical Subject Headings (MeSH) terms, "basal cell adenoma"[Mesh]) AND ("upper lip"[Mesh] OR "upper lip"[Mesh] OR "monomorphic adenoma"[Mesh]. The articles published between 1991 and December 2023 were included. Two authors methodologically assessed the titles and abstracts of the retrieved studies/case series/case reports, and any disparity was resolved by a third author. The references of all the included studies were manually checked to include any previously missed studies. The literature research revealed 14 cases of BCA originating from the minor salivary glands of the upper lip from 11 case reports/series [1,3-7,12,14,16-18] (Table 1).

**Table 1:** Summary of the reported cases of basal cell adenoma of the upper lip

Author (s) & year	Case no.	Age/Gender	Clinical findings	Histologic Pattern	Treatment & Recurrence
Margaritescu et al. 2005 [3]	1.	55/M	2x2 cm painless mass	Solid	-
	2.	57/F	1.5x2 cm painless mass	Solid	-
Minicucci et al. 2008 [7]	3.	51/F	4x4cm hard, painless	Solid	Excisional biopsy. No recurrence during the six-month follow-up
Antoniades et al. 2009 [16]	4.	68/M	Soft, well-demarcated, painless swelling	Solid	Excisional biopsy. No recurrence during the five-year follow-up
	5.	67/M	Soft, well-demarcated, painless swelling	Solid	Excisional biopsy. No recurrence during the 10-month follow-up
Soares et al. 2010 [12]		69/M	1.5 cm asymptomatic, purplish, well-demarcated, mobile, sub-mucosal nodule	trabecular	Surgical excision. No recurrence during the two-year follow-up
Kudoh et al. 2014 [1]	7.	59/M	1x1 cm soft, mobile, painless mass	Trabecular/Tubular	Excision. No recurrence
Gupta et al. 2014 [14]	8.	42/M	1x1 cm pale-pink, sessile, dome-shaped, painful swelling	Trabecular/Tubular	Excision.
Bhagat Singh et al. 2015 [4]	9.	28/F	-	Membranous	Total parotidectomy. No recurrence
	10.	42/F	-	Tubular	Local excision. No recurrence
Hayashi et al. 2015 [17]	11.	75/F	10 mm well-defined, painless, mobile sub-mucosal mass	Solid	Surgical excision. No recurrence during the 20-year follow-up
Beanes et al. 2015 [5]	12.	76/F	firm slow-growing, asymptomatic, firm, nodular lesion	tubular-trabecular	Excisional biopsy. No recurrence during the 8-year follow-up
Karim et al. 2015 [6]	13.	84/M	2 cm painless, mobile, firm, sub-mucosal mass	Trabecular-tubular	Surgical excision
Tatehara et al. 2019 [18]	14.	73/F	22 mm bluish-violet, elastic-hard, painless swelling	tubular or trabecular	Excisional biopsy. No recurrence during the 16-year follow-up
Present case	15.	48/F	1.2x1cm asymptomatic, firm, mobile mass	solid	Excisional biopsy. No recurrence during the one-year follow-up

In the 14 reported cases, there was no gender preponderance. Seven males [1,3,6,12,14,16], and seven females [3-5,7,17,18] presented with BCA of upper lip. The mean age of occurrence was 60.42±15.13 years, and the youngest and oldest patient in our review were 28 years [4], and 84 years [6] old respectively. The smallest and largest tumor mass was 10 mm [17], and 4 cm [7], respectively. All the lesions were painless and did not exhibit recurrence after surgical resection [1,3-7,12,14,16-18]. The longest follow-up was carried out for 20 years [17]. Histologically, the solid pattern was the predominant variant seen in six cases [3,6,7,17], followed by the tubular/trabecular pattern observed in five cases [1,5,6,14,18]. The membranous subtype was observed in only one case [4].

Our case findings are in coherence with the published literature. Our patient was a 48-year-old female who presented with an asymptomatic, mobile, firm, 1.2x1 cm soft tissue mass on the upper lip. Complete surgical excision of the tumor mass was carried out, and the solid subtype of BCA was confirmed on histopathological evaluation. The postoperative course was unremarkable, and no recurrence was observed after a one-year follow-up.

Despite the dearth of the reported cases of BCA of the minor salivary glands of the upper lip, BCA should always be given a place in the differential diagnosis of upper lip masses. The obscure clinical presentation, characterized by a slow-progressive asymptomatic upper lip mass underscores the importance of early identification, histopathological diagnosis, and therapeutic intervention. Difficulties such as delayed reporting and the potential for follow-up lapses emphasize the crucial need for healthcare professionals to recognize these implications, affecting both the health system and the patient's well-being.

Histopathological evaluation is widely recommended as the gold standard for a confirmatory diagnosis. While immunohistochemistry (IHC) examinations may lack specificity and are somewhat contingent on the histologic subtype of BCA, they can serve as an adjunct in differentiating these tumors [19]. The salient histopathological characteristics encompass monomorphic basaloid epithelial cell proliferation with a sparse cytoplasm and the lack of a chondromyxoid stromal matrix [15]. Based on their morphological characteristics, BCAs are divided into four patterns: solid, tubular, trabecular, and membranous, with the solid subtype being the most frequently observed [1,13]. 

Diagnosing BCA can be challenging as they mimic other lesions that also feature basaloid cells, such as pleomorphic adenoma (PA), adenoid cystic carcinoma (ACC), and basal cell adenocarcinoma (BCAC) [13,19,20]. The presence of a monomorphic basaloid epithelial pattern and lack of chondromyxoid stroma serve as histologic differentiating features between BCA and pleomorphic adenoma [1,15,19,21]. PA shows strong immunoreactivity for calponin and moderate for Pan CK and SMA indicating myoepithelial cell proliferation. It shows weak staining for Ki-67, which is a proliferative marker and mild staining in BCA [4,22].

The most challenging differential consideration is adenoid cystic carcinoma. BCAs typically display a well-circumscribed pattern and present numerous endothelial-lined channels. These characteristics contrast with the invasive pattern and the lack of vascularity observed in the microcystic regions of adenoid cystic carcinoma [19]. CD117(c-Kit) is a specific marker for adenoid cystic carcinoma and is negative for BCA [22].

The distinction between BCA and BCAC relies on the architectural differences despite the similarity in cellular composition. Unlike BCA, BCAC is a low-grade malignancy that demonstrates a non-encapsulated growth pattern, vascular or perineural invasion, cellular atypia, and increased mitotic activity [1,4,13,15,19]. Proliferative markers like Ki-67 and p63 are often used to confirm the histopathological diagnosis, as a strong positivity for Ki-67 and p63 are diagnostic for malignancy [22].

Pleomorphic adenoma is usually seen in younger individuals (<40 years), and the palate is the commonest intraoral site [4,23]. The probability of the mass being a mucocele may be negated by the site and age of the patient (mucoceles are more likely to occur on the lower lip of children and young adults) and a chronic duration of 3.5 years. The possibility of salivary duct cysts is highly unlikely as they characteristically appear as bluish, firm nodular swellings on the floor of the mouth and rarely on the lips [23,24]. The diagnosis of a malignant minor salivary gland tumor (adenoid cystic carcinoma and acinic cell carcinoma) may be ruled out based on the location, well-circumscribed mass, chronic duration, and lack of fixation [23,24].

Soft tissue infections of the lip (odontogenic/nonodontogenic) may be negated due to the asymptomatic and chronic duration of the lesion with normal dentition. Soft tissue lip abscess is generally acute, ill-defined, and fluctuant and exhibits inflammatory signs (pain and erythema) [23,24]. Lipomas are extremely rare in the oral cavity (1-4%) and characteristically manifest as a slow-growing, yellow-colored, soft, asymptomatic submucosal mass, with a typical “slip sign,” occurring mostly on the tongue and cheek region [23,24].

Hemangioma diagnosis may be negated taking into account the patient's age and gender (hemangiomas occur exclusively in infancy and young adults, especially in girls), chronicity of the lesion, non-compressibility, lack of a peculiarly bright red lesion, and a negative diascopy test [23,24]. Lymphangioma, a benign hamartomatous lesion of the lymphatic system, primarily affects infants and children and involves the tongue, although few lip cases have also been documented. The lip lesions frequently exhibit an asymmetric, asymptomatic, firm, and nodular pattern [23,24]. Leiomyoma, a benign smooth muscle tumor, infrequently occurs in the oral cavity due to the absence of smooth muscle in the region. The lesion mostly affects individuals in the 3rd decade and manifests as a slow-progressing, asymptomatic mass [24].

Reactive and neoplastic neural lesions (traumatic neuroma, neurofibroma, and schwannoma) may also be considered in the present case. The oral traumatic neuroma typically manifests as a nodular lesion, in close proximity to the mental foramen, tongue, or lips. However, the lesions are painful on palpation [24]. Schwannomas are benign neural tumors and manifest as discrete, firm, smooth-surfaced nodular lesions of the same color as the adjacent mucosa. They have a site affinity for the head and neck region, although lip involvement is an extremely rare occurrence [24]. Neurofibroma (NF) is a nerve sheath neoplasm, manifesting as a localized lesion or a component of the diffuse neurofibromatosis syndrome. However, solitary NF is an extremely unusual occurrence in the oral cavity (6% occurrence) and infrequently affects the lower lip [24]. 

The recommended treatment approaches include conservative techniques such as local resection or superficial gland removal, except in membranous cases where complete gland resection is required [25]. The recurrence rate in BCA is influenced by the histological pattern. While the solid and tubular-trabecular patterns exhibit an almost negligible recurrence rate, the membranous type displays a higher recurrence rate (24-37%), possibly due to its multi-centric nature [1,4,5,15,19,25]. Malignant transformation in BCA is infrequent, except in the membranous type, which exhibits a 4% transformation rate [5,13,16,18]. 

Recommendations

Basal cell adenoma is a distinct entity, with peculiar clinical and histological findings, which help in its diagnosis and guide the clinician to the correct treatment. Despite its rarity, BCA should be given a place in the differential diagnosis of upper lip masses. An early and accurate diagnosis of upper lip lesions is imperative, particularly because basal cell adenoma has the potential for malignant transformation.

## Conclusions

Basal cell adenomas are rare tumors, accounting for less than 3% of all salivary gland tumors. A precise diagnosis of BCA is crucial, as tumors with basal cells exhibit variations in treatment approaches and prognostic outcomes. Complete surgical resection exhibits a favorable outcome with a low recurrence rate.
